# A note of thanks to referees in 2012

**Published:** 2012

**Dementia & Neuropsychologia** finishes the sixth year of publication and we would like to thank our referees for sharing their expertise with us. The high quality of constructive criticisms and dedication of all our reviewers are highly appreciated by both the editors and the authors.

**Table:** 

Adriana Conforto (São Paulo, SP) Alessandro Ferrari Jacinto (São Paulo, SP) Alexandre Aluizio Costa Machado (São Paulo, SP) Alexandre Leopold Busse (São Paulo, SP) Antonio Lucio Teixeira Junior (Belo Horizonte, MG) Ariella Fornachari Ribeiro (Osasco, SP) Charles André (Rio de Janeiro, RJ) Claudia Sellitto Porto (São Paulo, SP) Claudio V. Mello (Portland, USA) Cleonísio Leite Rodrigues (Fortaleza, CE) Daniel Apolinário (São Paulo, SP) Denise Madeira Moreira (Rio de Janeiro, RJ) Eliane F. C. Banhato (Juiz de Fora, MG) Erasmo Barbante Casella (São Paulo, SP) Facundo F. Manes (Buenos Aires, Argentina) Florindo Stella (Rio Claro, SP) Francisco de Assis Carvalho do Vale (São Carlos, SP) Gabriel Rodriguez de Freitas (Rio de Janeiro, RJ) Gisela Tinone (São Paulo, SP) Helio Van Der Linden Junior (Goiânia, GO) Jacqueline Abrisqueta Gomez (São Paulo, SP) Jamary Oliveira Filho (Salvador, BA) José Luis de Sá Cavalcanti (Rio de Janeiro, RJ) José Renato Amaral (São Paulo, SP) Karolina Gouveia César (Taubaté, SP) Leonardo Ferreira Caixeta (Goiânia, GO) Leonardo Ierardi Goulart (São Paulo, SP) Leonel Tadao Takada (São Paulo, SP) Letícia Lessa Mansur (São Paulo, SP) Luciano de Gois Vasconcelos (Mogi das Cruzes, SP) Luis Felipe Berchielli (São Paulo, SP)	Magisetty Obulesu (Andhra Pradesh, India) Maira Okada de Oliveira (São Paulo, SP) Marcella Bellani (Verona, Italy) Marcelo Calderaro (São Paulo, SP) Marcelo E. Bigal (Whitehouse Station, New Jersey) Márcia Radanovic (São Paulo, SP) Marcio Luiz Figueredi Balthazar (Campinas, SP) Maria Niures Pimentel dos Santos Matioli (São Paulo, SP) Maria Sheila Guimarães Rocha (São Paulo, SP) Maria Teresa Carthery (Santo André, SP) Mariana E. Benassi-Werke (São Paulo, SP) Maurice Borges Vincent (Rio de Janeiro, RJ) Mirna Wetters Portuguez (Porto Alegre, RS) Mônica Sanches Yassuda (São Paulo, SP) Norberto Anizio Ferreira Frota (Fortaleza, CE) Paulo Henrique Ferreira Bertolucci (São Paulo, SP) Paulo Mattos (Rio de Janeiro, RJ) Paulo Pereira Christo (Belo Horizonte, MG) Rodrigo Rizek Schultz (São Paulo, SP) Roger Taussig Soares (São Paulo, SP) Rogerio Gomes Beato (Belo Horizonte, MG) Ronaldo Abrahan (Taubaté, SP) Sérgio Luís Blay (Rio de Janeiro, RJ) Sonia Maria Dozzi Brucki (São Paulo, SP) Tarso Adoni (São Paulo, SP) Thaís Helena Machado (Belo Horizonte, MG) Valéria Santoro Bahia (São Paulo, SP) Viviane Hiroki Flumignan Zétola (Curitiba, PR) Yára Dadalti Fragoso (Santos, SP) Ylmar Corrêa Neto (Florianópolis, SC)

